# The Bone-Forming Properties of Periosteum-Derived Cells Differ Between Harvest Sites

**DOI:** 10.3389/fcell.2020.554984

**Published:** 2020-11-25

**Authors:** Lisanne C. Groeneveldt, Tim Herpelinck, Marina Maréchal, Constantinus Politis, Wilfred F. J. van IJcken, Danny Huylebroeck, Liesbet Geris, Eskeatnaf Mulugeta, Frank P. Luyten

**Affiliations:** ^1^Prometheus, Division of Skeletal Tissue Engineering, KU Leuven, Leuven, Belgium; ^2^Skeletal Biology and Engineering Research Center, KU Leuven, Leuven, Belgium; ^3^OMFS IMPATH Research Group, Department of Imaging and Pathology, KU Leuven, Leuven, Belgium; ^4^Oral and Maxillofacial Surgery, University Hospitals Leuven, Leuven, Belgium; ^5^Department of Cell Biology, Erasmus University Medical Center, Rotterdam, Netherlands; ^6^Center for Biomics, Erasmus University Medical Center, Rotterdam, Netherlands; ^7^Department of Development and Regeneration, KU Leuven, Leuven, Belgium; ^8^Biomechanics Research Unit, GIGA-R In Silico Medicine, Université de Liége, Liège, Belgium; ^9^Biomechanics Section, KU Leuven, Leuven, Belgium

**Keywords:** cell differentiation, mandible, maxilla, mesenchymal stromal cells, osteogenesis, periosteum, tibia, transcriptome

## Abstract

The development of alternatives for autologous bone grafts is a major focus of bone tissue engineering. To produce living bone-forming implants, skeletal stem and progenitor cells (SSPCs) are envisioned as key ingredients. SSPCs can be obtained from different tissues including bone marrow, adipose tissue, dental pulp, and periosteum. Human periosteum-derived cells (hPDCs) exhibit progenitor cell characteristics and have well-documented *in vivo* bone formation potency. Here, we have characterized and compared hPDCs derived from tibia with craniofacial hPDCs, from maxilla and mandible, respectively, each representing a potential source for cell-based tissue engineered implants for craniofacial applications. Maxilla and mandible-derived hPDCs display similar growth curves as tibial hPDCs, with equal trilineage differentiation potential toward chondrogenic, osteogenic, and adipogenic cells. These craniofacial hPDCs are positive for SSPC-markers CD73, CD164, and Podoplanin (PDPN), and negative for CD146, hematopoietic and endothelial lineage markers. Bulk RNA-sequencing identified genes that are differentially expressed between the three sources of hPDC. In particular, differential expression was found for genes of the HOX and DLX family, for *SOX9* and genes involved in skeletal system development. The *in vivo* bone formation, 8 weeks after ectopic implantation in nude mice, was observed in constructs seeded with tibial and mandibular hPDCs. Taken together, we provide evidence that hPDCs show different profiles and properties according to their anatomical origin, and that craniofacial hPDCs are potential sources for cell-based bone tissue engineering strategies. The mandible-derived hPDCs display - both *in vitro* and *in vivo -* chondrogenic and osteogenic differentiation potential, which supports their future testing for use in craniofacial bone regeneration applications.

## Introduction

The development of effective treatments for large bone defects incurred by trauma, osteonecrosis or tumor resection remains a challenge, which is further complicated by factors such as infections and congenital abnormalities. Currently, autografts are considered as the standard treatment of these bone defects, however, this approach requires large volumes of bone. In addition, it also requires a second surgical site where donor tissue is harvested, thereby introducing donor site morbidity (including pain) and potential major complications (in up to 10% of the cases; Arrington et al., 1996). These include vascular injuries or deep infection in the donor site, and the possibility of herniation of abdominal contents in case an iliac crest graft is used. Furthermore, usually these donor sites provide insufficient quantities of tissue for filling large bone defects and, in addition, poor vascularization of the tissue graft can lead to necrosis (Meijer et al., 2008).

Bone tissue engineering presents an opportunity for the repair of large bone defects, with the engineered constructs classically being composed of appropriate cells that are able to proliferate and differentiate, and growth factors to instruct cell differentiation in a 3D environment that provides some biomechanical support (Leijten et al., 2015). Skeletal stem and progenitor cells (SSPCs), often referred to as mesenchymal stromal cells (MSCs), whereas these originate from various tissues including bone marrow, adipose tissue, gingiva, dental pulp and the periosteum, are being assessed for their potential in bone tissue engineering. Recently, a *bona fide* human skeletal stem cell characterized by a PDPN^+^ CD146^–^ CD73^+^ CD164^+^ signature was identified (Chan et al., 2018). This stem cell population demonstrates significant amplification properties in response to fracture. Similarly, periosteum-derived cells (PDCs) undergo a rapid expansion to assist in callus formation (Colnot et al., 2012), suggesting the presence of a stem or progenitor cell population among PDCs.

Depending on their tissue of origin, differences in the properties of SSPCs regarding cellular proliferation, differentiation and senescence, as well as cytokine release and gene/mRNA expression profiles have been documented (Dominici et al., 2006; Kern et al., 2006; Tsai et al., 2007). Although bone marrow-derived SSPCs (BM-SSPCs) are osteogenic *in vitro* and form bone tissue *in vivo*, the bone forming cells in a long bone fracture forming the callus are predominantly PDCs (Colnot et al., 2012). Furthermore, periosteum forms a larger callus with a higher mineral content compared to bone marrow (Guichet et al., 1998), and cells in the periosteum display higher proliferation following injury (Brighton and Hunt, 1997). Ultimately, some data suggests that PDCs contain a higher bone regenerative potential than BM-SSPCs (Duchamp de Lageneste et al., 2018). For these reasons PDCs are increasingly studied as a clinically relevant source for bone tissue engineering purposes (Nakahara et al., 1991; De Bari et al., 2006; van Gastel et al., 2012; Owston et al., 2019; Bolander et al., 2020).

The anatomical location and other types (e.g., developmental molecular signatures) of positional information are important factors when studying *in vivo* bone formation (Leucht et al., 2008). During development, facial cartilage and bone are predominantly derived from cranial neural crest, unlike long bones, such as the tibia, that originate from limb skeletal precursors derived from the somatic layer of the lateral plate mesoderm (Noden, 1982; Couly et al., 1993). In the neural plate stage embryo, neural crest and placodal cells develop in the future cranial region at the border of the neural plate with the non-neural ectoderm under the influence of FGF signaling together with precisely dosed Wnt and/or BMP signaling. The actions of these signals converge on specific gene families of Msh-type homeobox (MSX1, MSX2), distal-less homeobox (DLX) and paired domain (PAX) transcription factors (Villanueva et al., 2002; Tribulo et al., 2003; Meulemans and Bronner-Fraser, 2004; Green et al., 2015). The cranial neural crest cells extensively migrate to the pharyngeal arches and form multiple ectomesenchymal cell types including thymus cells, odontoblasts, osteoblastic, adipogenic and chondrogenic cells, muscle cells and neuronal ganglia (Green et al., 2015). Craniofacial bone tissue has a higher turnover rate, “ages” more slowly, and has higher levels of osteoblastic markers compared to appendicular skeletal bone tissue such as that from tibia (Aghaloo et al., 2010). Furthermore, substantial differences in structure and bone biology exist between maxillary and mandibular bone tissue. Maxillary bone tissue arises by intramembranous ossification and consists of trabecular bone surrounded by a thin cortical layer, has low bone density and mineral content, and is highly vascularized. Mandibular bone tissue develops by both endochondral ossification and intramembranous ossification. It consists of lamellar bone tissue that is surrounded by a thick cortical layer, has a high mineral content and bone density, and shows more centralized vascularization (Park et al., 2008; Lindhe et al., 2013). In a first step to assess the implications of these differences on tissue regeneration strategies, Leucht et al. (2008) performed cross-grafting of tibial periosteum onto craniofacial defects and vice versa, and further have shown that embryonic origin and homeobox gene expression influenced the regeneration potential. Despite these well-known differences in bone origin, structure and biology – and their effect on tissue regeneration strategies – many bone tissue engineering therapies intended for use in maxillofacial context continue using the same cell source as their long bone counterparts.

In this study, we built on the work of Leucht et al. (2008), toward clinical translation. We investigate the molecular differences of *in vitro* expanded periosteal cells from maxilla and mandibula in the context of their potential use as cell source for maxillofacial tissue engineering applications, using tibial hPDCs as benchmark. Cell surface markers, growth curves, trilineage differentiation potential, gene/mRNA expression and *in vivo* bone formation were assessed. We show that craniofacial PDCs meet essential criteria of SSPCs (Chan et al., 2018). In addition, *in vitro* differentiation and the mRNA profiling by bulk RNA-sequencing (RNA-seq) show that hPDCs exhibit different properties depending on their retrieval site.

## Materials and Methods

### Isolation and Culture of hPDCs

Periosteal samples (5 × 10 mm) were obtained from the posterior areas of the maxilla and mandible of 16- to 30-year-old healthy patients who underwent bimaxillary orthognathic surgery, after informed consent was obtained (Belgian registration number B322201731127). The tibial samples were harvested by orthopedic surgeons as described previously (Eyckmans and Luyten, 2006). These samples were digested in medium consisting of Dulbecco’s modified Eagle’s medium (DMEM) supplemented with 10% Fetal Bovine Serum (FBS; Thermo Fisher Scientific, Waltham, MA, United States) and 1% antibiotics-antimycotics solution (100 units penicillin/ml, 100 μg streptomycin/ml and 0.25 μg amphotericin-B/ml; Invitrogen), from here on referred to as DMEM-complete (DMEM-C), in addition of 4,400 units per 10 ml Collagenase type-IV (Gibco) under constant agitation for 16 h. Cells were from then on cultured in DMEM-C. Population doublings (PD) of these human PDCs (hPDCs) were calculated upon passaging using the formula log_2_(n_2_)−log_2_(n_1_), with n_1_ representing the number of cells seeded in the dish/flask and n_2_ the number of cells obtained after trypsinization (TrypLE, Thermo Fisher Scientific) of cells from the same dish/flask at 90% confluency.

### Cellular Senescence

For detection of senescent cells, 10^5^ cells were plated per well in a 6-well plate in DMEM-C. After one day, the cells were fixed using 4% paraformaldehyde (PFA) for 5 min, rinsed using PBS and stained with 2 ml/well filtered (0.22 μm; Millex-GP) staining solution consisting of 36 mM citric acid, pH 6.0 (Sigma Aldrich), 4.5 mM potassium ferricyanide (Merck), 0.14 M NaCl, 1.8 mM MgCl_2_ (Merck) and 1 mg/ml X-gal (Biotium). After 6 h, the cells were imaged using a Primovert inverted microscope (Zeiss).

### Cell Proliferation

For detection of proliferating cells, staining for incorporated EdU was performed using the Click-iT^TM^ EdU Alexa Fluor^TM^ 555 Imaging Kit (Thermo Fisher Scientific) according to the manufacturer’s protocol. In short, 10^5^ cells/well were seeded in a 6-well plate in DMEM-C. The following day, medium was replaced with DMEM-C containing 10 μM EdU, followed by an incubation of 72 h. Next, cells were fixated using 3,7% formaldehyde in PBS (VWR) for 15 min, rinsed twice with 3% bovine serum albumin (BSA, Sigma Aldrich) in PBS and lysed with 0.5% Triton X-100 in PBS (Sigma Aldrich) for 20 min. This was followed by adding the Click-iT reaction cocktail for 30 min in absence of light, quick washing steps of 1 min using subsequently 3% BSA dissolved in PBS and later on in PBS, addition of the Hoechst solution for 30 min in a 1:2,000 dilution in PBS and final washing steps using PBS for 1 min.

### Cell Metabolism

Cell metabolism was measured by using 3-(4,5-dimethylthiazol-2-yl)-2,5-diphenyltetrazolium bromide (MTT) assay. Living cells contain the mitochondrial reductase enzyme, which reduces MTT toward formazan forming salt crystals. By measuring the amount of reduction of MTT toward formazan, the cell metabolism was measured. This was done by removing remaining MTT and solubilizing of the salt crystals in order to get with the different quantities of salt crystals corresponding differences in color intensity (Mosmann, 1983). Tests were performed as technical replicates 6 times per donor at 6, 9, and 12 weeks of culture. 10,000 cells were seeded in a 24-well plate in 0.5 ml DMEM-C. After 1 day, DMEM-C was replaced with DMEM containing 250 μg MTT/ml (Sigma Aldrich). After 4 h, medium and remaining MTT were removed and 1 ml absolute ethanol was added to each well in order to solubilize the salt crystals. These solutions were transferred to a 96-well plate and absorbance was read at 570 and 670 nm with a Synergy HT reader (BioTek). In order to calculate the metabolic rate, the background absorbance (at 670 nm) was subtracted from the absorbance at 570 nm and corrected for the blank values which were obtained by performing all steps on wells containing no cells.

### Flow Cytometry

10^6^ cells were at passage 6 washed in 500 μl PBS + 1% FBS and incubated at the following dilutions with anti-CD90-APC (1:250), anti-CD164-PE (1:250), anti-CD45-FITC (1:1,000), anti-CD31-FITC (1:1,000), anti-CD235ab-FITC (1:1,000; all from BioLegend), anti-CD146-BV711 (1:250), anti-CD73-BV395 (1:250) and anti-PDPN-BV510 (1:100; all from BD Bioscience) in 200 μl PBS + 1% FBS for 15 min in a dark area. The cells were then washed twice with 1% FBS in PBS. For compensation controls, UltraComp eBeads Compensation Beads (Thermo Fisher Scientific) were used. Gating was performed using Fluorescence-Minus-One (FMO) controls. Fluorescence measurement was performed on a FACS Calibur (BD Biosciences) using 100,000 events.

### Differentiation of hPDCs

Human periosteum-derived cells were primed for adipogenic, osteogenic and chondrogenic differentiation for 14, 21, and 7 days, respectively. Analysis was performed by staining with Oil Red O, Alizarin Red and Alcian Blue, respectively.

For adipogenic differentiation of hPDCs, cells were cultured in 24-well plates at a density of 19,000 cells/well in 0.5 ml DMEM-C. After 2 days, the medium was changed for adipogenic differentiation medium, i.e., Minimum Essential Medium Eagle - alpha modification (α-MEM; Invitrogen supplemented with 10% FBS, 1% antibiotics-antimycotics solution, 1 μM dexamethasone + 10 μg/ml human insulin (Sigma Aldrich), 100 μM indomethacin (Sigma Aldrich) and 25 μM 3-isobutyl-1-methylxanthine (Sigma Aldrich). The cells were cultured in a humidified atmosphere at 5% CO2 and 37°C for 14 days and medium was replaced every 2 days. Lipid droplets were stained by Oil Red O. Cells in monolayer were washed in PBS and fixed for 30 min at room temperature in 10% formaldehyde (VWR) diluted in PBS. Next, cells were washed with Milli-Q water and rinsed in 60% isopropanol (VWR) diluted in Milli-Q water. Cells were allowed to dry completely and then stained with 0.2% Oil Red O (Sigma Aldrich) in Milli-Q water for 60 min. These cells were then washed with Milli-Q water before obtaining pictures using the Primovert microscope (Zeiss).

For osteogenic differentiation of hPDCs, cells were cultured in 24-well plates at a density of 8,550 cells per well in 0.5 ml DMEM-C, first allowing them to proliferate. After 1 day, medium was then changed for osteogenic medium consisting of DMEM-C supplied with 100 nM dexamethasone (Sigma Aldrich), 10 mM β-glycerophosphate disodium salt hydrate (Sigma Aldrich) and 50 μg/ml L-ascorbic acid 2-phosphate (Sigma Aldrich). Cells were cultured in a humidified atmosphere at 5% CO_2_ and 37°C for 21 days, during which this medium was replaced every 3 days. Cells in monolayer were washed in PBS, fixed with methanol at −20°C (Fisher Scientific), stored for 1 h at 4°C, and washed again with PBS. Cells were stained for 1 h with a 2% Alizarin-S stain (Sigma Aldrich) in Milli-Q water with pH adjusted to 4.2 (Merck) while the plate was placed on a shaker for constant agitation. After staining, cells were washed with Milli-Q water. Pictures were made using a Discovery.v8 SteREO microscope (Zeiss).

For chondrogenic differentiation of hPDCs, the cells were cultured in micromasses. Micromasses containing 200,000 cells in 10 μl DMEM-C after centrifugation at 260 RCF for 10 min were added to individual wells of 24-well plates. After 2 h, an extra 0.5 ml of DMEM-C was added to each well. After 1 day, this medium was replaced by chondrogenic inductive medium (DMEM-F12 (Invitrogen) containing 2% FBS, antibiotics-antimycotics solution, 1x ITS + premix universal culture supplement (Corning), 100 nM dexamethasone, 10 μM Y27632 (Axon Medchem), 50 μg/ml L-ascorbic acid 2-phosphate, 40 μg/ml proline (Sigma Aldrich) and 10 ng/ml transforming growth factor-β1 (TGFβ1; PeproTech). These cells were cultured under humidified conditions at 37°C and 5% CO_2_ for 7 days; medium was replaced every 2 days. Sulfated glycosaminoglycans in the micromasses were stained by Alcian Blue (Roth). The cells were rinsed with PBS and fixed with excess ice-cold methanol for 1 h at 4°C. This methanol was removed and cells were rinsed with Milli-Q water prior to adding 0.1% Alcian Blue staining solution (in 0.1 M HCl at pH 2.4) for 1 h on a shaker. Alcian Blue stain was removed and the cells were rinsed with Milli-Q water before pictures were made using the Discovery.v8 SteREO microscope (Zeiss). At days 1 (control), 3, 5 and 7, micromasses were also obtained for confirmation of chondrogenic differentiation by RT-qPCR. Micromasses were individually lysed in 350 μl RLT buffer (Qiagen) containing 1% β-mercaptoethanol (Sigma Aldrich) and homogenized by thoroughly pipetting up and down. Samples were directly placed on ice and frozen at −80°C until further processing.

### RNA Isolation, cDNA Synthesis and Quantitative PCR

RNA was isolated from biological duplicates when a cumulative PD∼10 was reached for RT-qPCR and at PD∼16 for RNA-seq analysis. 10^6^ cells were rinsed twice in PBS and centrifuged at 260 RCF for 10 min. After removing the supernatant, the cell pellet was lysed by adding 350 μl RLT buffer (Qiagen) containing 1% β-mercaptoethanol (Sigma Aldrich) and homogenized by thoroughly pipetting up and down. Samples were directly placed on ice and frozen at −80°C until further processing. The Qiagen RNeasy mini kit was used to isolate RNA according to the manufacturer’s instructions. cDNA was obtained by reverse transcription of 500 ng of total RNA, using the RevertAid H Minus First Strand cDNA Synthesis Kit, 1 mM oligo (dT)20 and random hexamer primers (Thermo Fisher Scientific).

RT-qPCR was performed on expanded cells without stimulation for differentiation for RNA encoding alkaline phosphatase (*ALPL*), osteocalcin (*BGLAP*), osteopontin (*SPP1*), peroxisome proliferator activated receptor gamma (*PPARG*), collagen type-X (*COL10A1*), SRY (sex determining region Y)-box 9 (*SOX9*) and vascular endothelial growth factor receptor 1 (*VEGFR*-1). On chondrogenically differentiated cells, the expression of aggrecan (*ACAN*), *Col10A1* and Runt-related transcription factor 2 (*RUNX2*) were defined. Samples were processed and measured in technical duplicates on a Step-One-Plus PCR machine (Applied Biosystems) using a Sybr Green detection system (Life Technologies) according to the manufacturer’s instructions, and normalized using glyceraldehyde 3-phosphate dehydrogenase (*GAPDH*) as a housekeeping gene. The respective primer sequences are listed in the Supplementary Table S1.

### RNA-Sequencing and Data Analysis

RNA-seq samples were obtained from three donors for the maxillary samples (HP417, HP421, and HP424), four donors for mandibular samples (HP418, HP422, HP425, and HP427), and three tibial donors (HP415, HP420, and HP423). RNA-seq samples were prepared from hPDCs at cumulative PD∼16 in technical duplicates (A and B) and cDNA libraries were generated using the Illumina TruSeq Stranded mRNA Library Prep Kit. These libraries were then sequenced (50 bp length) according to the Illumina TruSeq Rapid v2 protocol in 5 runs, 2 lanes each run, on an Illumina HiSeq 2500 sequencer. Low quality reads and contaminants (e.g., sequence adapters) were removed using Trimmomatic (Bolger et al., 2014). Sequences that passed the quality assessment were aligned to the GRCh38 genome using HiSat2 (version 2.1.0) (Kim et al., 2015). Transcript abundance level (transcript count) was generated using HTSeq (version 0.9.1) (Anders et al., 2015). The transcript counts were further processed using R (version 3.4.0). Data normalization and removal of batch effect was performed using the EDASeq R package (Risso et al., 2011) and RUVseq package (Remove Unwanted Variation from RNA-Seq package). Differential expression analysis was performed using the edgeR R package (Robinson et al., 2010), using the negative binomial generalized linear model approach. Differentially expressed genes with false discovery rate (FDR) ≤ 0.05 after Benjamini-Hochberg correction for multiple testing and expression level greater than 1 count per million (CPM) were retained and used for further processing, gene ontology and pathway analysis. Gene enrichment analysis was performed using metascape (Zhou et al., 2019). The RNA sequencing data is deposited at NCBI with GEO accession number GSE149167.

### *In vivo* Bone Formation

After proliferation, 10^6^ cells suspended in 25 μl of DMEM-C were seeded on NuOss scaffolds (21 mm^3^; ACEuropa). One hour after seeding, an additional 5 ml of DMEM-C was added. After overnight incubation at 37°C and 5% CO2, scaffolds were randomly implanted subcutaneously over the available mice in triplicates with a maximum of 4 for each mouse at the shoulder and the back in the limb region of 8-week-old female NMRI^*nu/nu*^ mice (Janvier). All animal procedures were approved by the local ethical committee for Animal Research (KU Leuven). The animals were housed according to the guidelines of the Animal Facilities at Leuven (KU Leuven). Explants were harvested after 8 weeks.

Bone formation was analyzed using the Phoenix NanoTom S CT system (GE measurement) at a voltage of 60 kV and a current of 200 mA. An aluminium filter of 1 mm was used to filter out the low energetic radiation. CTAn and Batch Manager Software programs (Bruker micro-CT, Kontich, Belgium) were used for 3-D quantification of the bone volume and calcium phosphate (CaP) grains of the NuOss scaffold. A three-level automatic Otsu segmentation algorithm was used to segment the newly formed bone tissue and CaP-grains from the background in combination with manually chosen thresholds. Noise was reduced by removing black and white speckles smaller than 200 voxels and a closing operation of 2 voxels was performed to solidify the resulting structure, providing binarized images suitable for the 3-D analysis. Percentage of bone was calculated with respect to total explant volume (Kerckhofs et al., 2013). After decalcification in EDTA, dehydration through a series of graded ethanol baths, embedding in paraffin and sectioning, histological stainings were performed. Staining with hematoxylin and eosin (H&E) was done by a dip in Hematoxylin-solution for 2 min, followed by a wash in H_2_O and subsequently a staining in 1% Eosin (Klinipath, Duiven, Netherlands) for 7 min (Bolander et al., 2016). For Alcian Blue staining, samples were rehydrated, stained for 30 min in 0.5% Alcian Blue (Roth) solution in 1 M HCl (pH = 1). Nuclear Fast Red (Vector) was used for staining of the nuclei during 5 min. For staining with Safranin O, samples were dipped in acidic alcohol (1% HCl in 70% ethanol). Next, they were placed in Fast Green (Klinipath) for 3 min, dipped in 1% acetic acid and then stained in 0.1% Safranin O (Sigma Aldrich) for 10 min. Masson’s trichrome staining was performed using Bouin’s solution (Sigma Aldrich) as mordant followed by 5-minute-dips in Weigert’s Iron Hematocxylin Solution, Biebrich Scarlet-Acid Fuchsin, Phosphotungstic/Phosphomolybdic Acid Solution and Aniline Blue Solution (all from Sigma Aldrich), followed by a dip in 1% acetic acid for 2 min. The sections were cleared in HistoClear (National-Diagnostics) and mounted in Pertex (Histolab). Pictures were made using the IX83 microscope (Olympus).

### Statistical Analysis

Statistical analysis for cellular senescence, quantitative PCR and *in vivo* bone formation was performed using the Kruskal-Wallis test. In case *p* < 0.05 for Kruskal-Wallis, a two-sided Wilcoxon test was performed to make comparisons for the qPCR validation of the RNA-seq data and to analyze cellular senescence, proliferation and metabolism rate. Student’s *t*-test was used to make comparisons for the chondrogenically differentiated micromasses. The Bonferroni correction was used to correct for multiple testing. All statistical analyses were performed in R (version 3.6.0). Results were considered statistically significant at *p* < 0.05. Data are presented as mean values ± standard deviation (SD) or 95% confidence interval (CI) when stated.

## Results

### Characterization of the Isolated and Expanded Human Periosteum-Derived Cells

**FIGURE 1:**
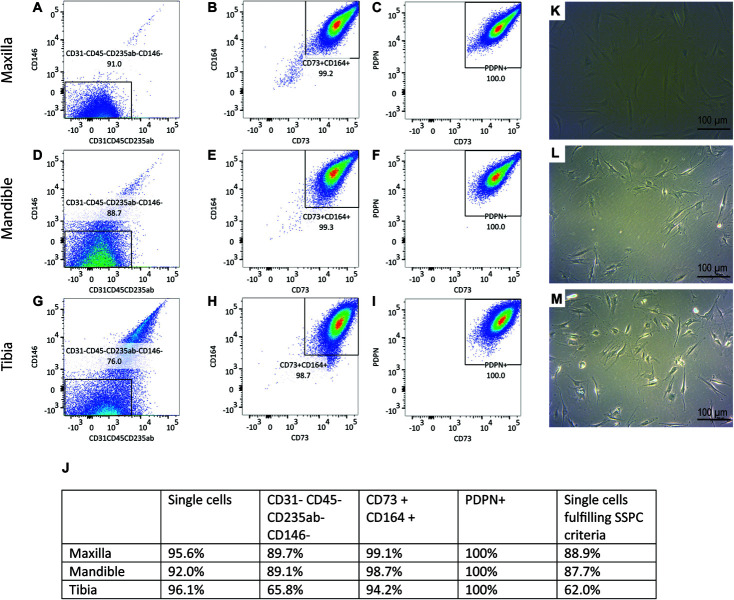
**(A)** Flow-cytometric analysis of periosteal-derived cells obtained from maxilla **(A–C)**, mandible **(D–F)** and tibia **(G–I)**. Gating is performed using FMO’s for each run. *n* = 3. 100.000 events were read for each sample. **(J)** Summary of flow-cytometric analysis, *n* = 3 for each origin. SA-β-gal staining on periosteal cells derived from maxilla **(K)**, mandible **(L)**, and tibia **(M)** demonstrating no precipitation of X-gal.

Flow cytometric analysis was performed on culture expanded hPDCs to confirm the presence of SSPCs (Figures 1A–I). 88.9% of the maxillary hPDCs were positive (+) for CD73, CD164 and PDPN, while negative for CD31, CD45, CD235ab, and CD146 (Figure 1J). In contrast, 87.7% of the mandibular cells and 62.0% of the tibial hPDCs met the SSPC criteria. All cells were selected based on their capacity to adhere to plastic (Figures 1K–M).

**FIGURE 2:**
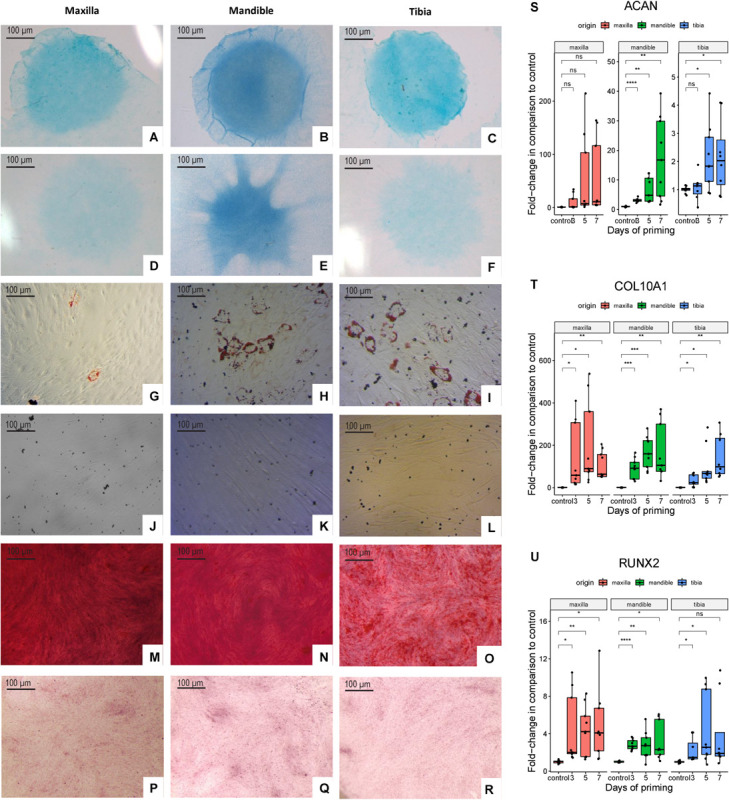
Alcian Blue staining of chondrogenically differentiated hPDCs obtained from maxilla, mandible and tibia **(A–C)** and cells cultured in DMEM-C **(D–F)**. Oil Red O staining of adipogenically differentiated hPDCs **(G–I)** and cells cultured in DMEM-C as controls **(J–L)**. Cells stained with Alizarin Red staining to detect calcium phosphate deposition of osteogenically differentiated hPDCs **(M–O)** and controls cultured in DMEM-C **(P–R)**. RT-qPCR performed at days 1 (control), 3, 5 and 7 for the chondrogenic genes *RUNX2*, *COL10A1* and *ACAN* showed as fold-change compared to the control after correction for GAPDH, *n* = 3 in biological triplicates. *p ≤ 0.05, **p ≤ 0.01, ***p ≤ 0.001, ****p ≤ 0.0001 **(S–U)**.

Alcian Blue-positive micromasses from maxillary and mandibular and tibial hPDCs indicated their ability to differentiate toward the chondrogenic lineage (Figures 2A–C; controls in Figures 2D–F). Adipogenic differentiation of hPDC cultures in monolayer was verified by staining with Oil Red O, for hPDCs obtained from the three different origins (Figures 2G–I; controls in Figures 2J–L). Calcium phosphate formed by osteogenic primed cells in monolayer was stained by Alizarin Red (Figures 2M–O; controls in Figures 2P–R) and showed mineral deposition by the hPDCs, independent of their origin. Additional RT-qPCR on chondrogenically differentiated micromasses at days 1 (control), 3, 5, and 7 showed that *RUNX2* (Figure 2U) and *COL10A1* (Figure 2T) were expressed at higher levels after chondrogenic differentiation in comparison to undifferentiated micromasses at day 1. Also *ACAN* was expressed from day 5 at statistically significantly higher levels compared to the controls (Figure 2S). These results indicated that plastic-adherent hPDCs derived from maxilla and mandible could be expanded, and that these cultured hPDCs were positive for acknowledged SSPC surface markers and able to differentiate toward three different cell types.

### Human PDCs Can Be Expanded for 2 Months

**FIGURE 3:**
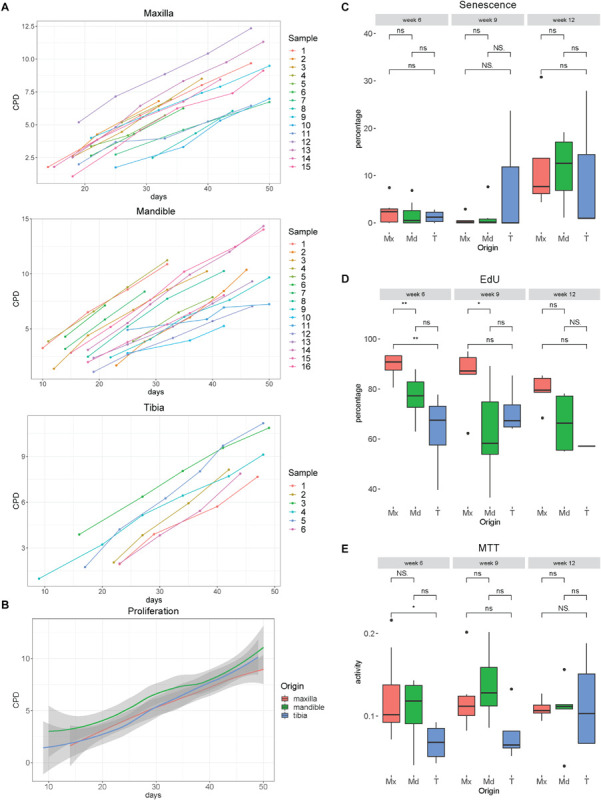
**(A)** Proliferation curves of hPDCs obtained from maxilla, mandible and tibia, wherein the X-bar displays the time in days and the vertical axis the cumulative population doublings (CPD). **(B)** Mean proliferation curves of hPDCs from maxilla (red), mandible (green) and tibia (blue), with confidence interval showed in gray. **(C)** Percentage of SA-β-gal staining-positive cells at 6, 9 and 12 weeks of expansion in DMEM-C. **(D)** Proliferation-capacity of hPDCs showed by the percentage of EdU-positive cells after 6, 9, and 12 weeks of expansion in DMEM-C. **(E)** Metabolic activity shown by MTT-test of hPDCs obtained from maxilla (Mx), mandible (Md) and tibia (T) after 6, 9 and 12 weeks of expansion in DMEM-C. *p ≤ 0.05, **p ≤ 0.01.

We have tested the ability to expand hPDCs obtained from maxilla versus mandible and tibia. The proliferation rates of hPDCs during *in vitro* expansion under the culture conditions used, measured in cumulative population doublings (CPDs), were similar and independent of their origin (Figures 3A,B). After 6, 9 and 12 weeks of expansion, staining with X-gal for senescence-associated β-Galactosidase was performed. During the first 9 weeks the average rates of such cells remained below 5%. After 12 weeks significantly higher rates were found, up until 28% of the cells (*p* = 0.045; Figure 3C). Quantification of proliferation was measured by staining for incorporated EdU, again after expansion for 6, 9 and 12 weeks. About 75% of the cells were EdU + after 6 and 9 weeks of expansion (Figure 3D) with a significantly higher proliferation rate for maxillary hPDCs after 6 weeks in comparison to mandibular and tibial hPDCs.

Assessing cellular metabolic activity using the MTT colorimetric assay, we saw a trend toward lower mitochondrial metabolic rates in tibia-derived hPDCs, however, no statistically significant differences were found between the maxillary, mandibular and tibia-derived hPDCs (Figure 3E). Furthermore, no decline in the metabolic activity was observed after 12 weeks. From this, we conclude that hPDCs, regardless of their tissue source, can be expanded during at least 9 weeks, with an expected yield of about 350 million cells after 50 days of proliferation. This yield is sufficient for typical applications in bone tissue engineering (Lammens et al., 2020).

### *In vitro* Analysis of Marker Genes on Expanded hPDCs From 3 Different Origins

**FIGURE 4:**
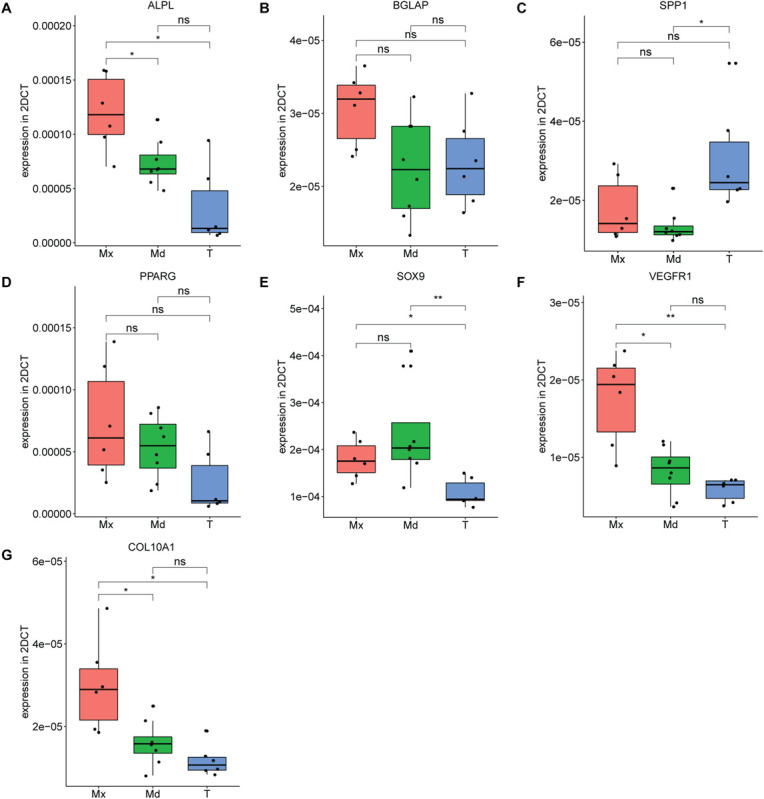
Expression level of ALPL **(A)**, BGLAP **(B)**, SPP1 **(C)**, PPARG **(D)**, SOX9 **(E)** VEGFR-1 **(F)** and COL10A1 **(G)** of hPDCs from maxilla (Mx), mandible (Md) and tibia (T) after expansion in DMEM-C at PD = 10, *n* = 3 in biological duplicates for each origin corrected for GAPDH. *p ≤ 0.05, **p ≤ 0.01, ***p ≤ 0.001, ****p ≤ 0.0001.

In order to investigate further the differences in mRNA profiles between the respective *in vitro* expanded hPDCs, we performed RT-qPCR on acknowledged marker genes for bone. These include *ALP*, a marker of osteogenic differentiation; *BGLAP*, coding for a protein secreted in bone solely by osteoblasts; and *SPP1*, a gene expressed by multiple cell types including osteoblasts and osteoclasts. The steady-state transcripts of *ALP* are higher in maxillary compared to mandibular (*p* = 0.025) and tibial hPDCs (*p* = 0.0013) (Figure 4A), whereas *BGLAP* transcripts showed no statistically significant differences (Figure 4B). *SPP1* was upregulated for tibia-derived cells when compared to hPDCs from mandible (*p* = 0.014; Figure 4C). For the adipogenic receptor *PPARG* no differences were seen that are statistically significant (Figure 4D). *SOX9* was upregulated in both maxillary and mandibular cells compared to tibial hPDCs (*p* = 0.03 and *p* = 0.008, respectively; Figure 4E). *VEGFR-1* transcripts were higher in maxillary compared to tibial (*p* = 0.007) and mandibular hPDCs (*p* = 0.04) (Figure 4F). Also for *COL10A1* an upregulation was seen for maxillary hPDCS compared to mandibular and tibial hPDCs (*p* = 0.016 and 0.013, respectively, Figure 4G).

### Bulk RNA-Seq of Cultured Adult hPDCs Reveals Differences That Reflect Different Embryonic Origins

**FIGURE 5:**
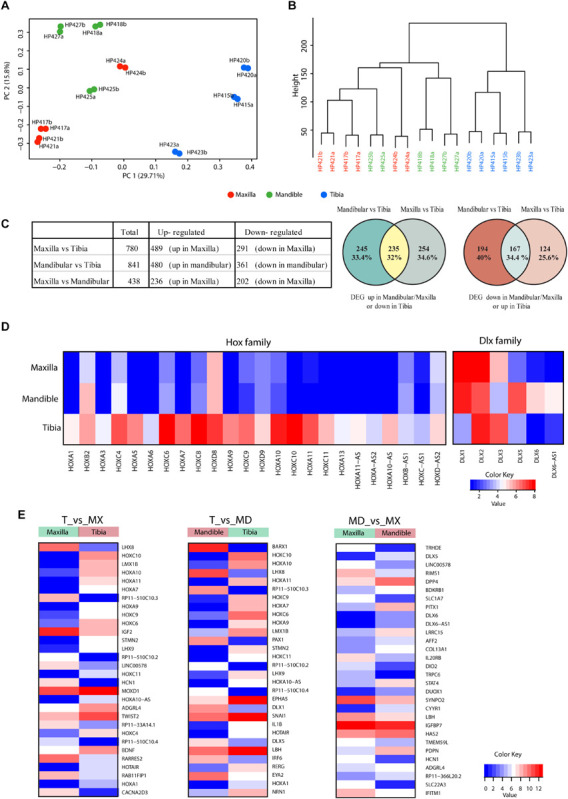
**(A)** Principal component analysis of RNA-sequencing on hPDCs obtained from maxilla (red), mandible (green) and tibia (blue). Each dot represents the sample mean from a single donor. **(B)** Cluster dendrogram based on RNA-sequencing results. **(C)** Table showing the number of differentially expressed genes and venn diagrams of differentially expressed genes between maxillary, mandibular and tibial hPDCs based on RNA-seq. **(D)** Heatmap of Hox and DLX family genes based on RNA-seq data. **(E)** Heatmap of the 30 most differentially expressed genes in the comparisons maxilla (Mx) vs. tibia (T), mandible (Md) vs. T and Mx vs. Md.

We have also performed bulk RNA-seq on expanded cells. Principal component analysis (PCA; Figure 5A) and the hierarchical clustering (dendrogram in Figure 5B) show that the differences in anatomical origin of the hPDCs are reflected in bulk mRNA expression differences. To capture the genes that contribute to this distinction, we have performed differential gene expression analysis between the hPDCs. 780 differentially expressed genes (DEGs; FDR < 0.05, log fold change > 1) were identified between tibial and maxillary hPDCs. 291 of these were down-regulated and 489 up-regulated in maxilla-derived hPDCs. Between mandible and tibia-derived hPDCs 841 DEGs were identified, with 361 genes down- and 480 genes up-regulated in mandibular hPDCs (Figure 5C and Supplementary Tables S2, S3). In these comparisons only around 33% of the DEGs are common between maxilla and mandible derived hPDCs compared to tibial hPDCs, attesting to the large difference that exists between maxilla and mandible-derived hPDCs. Finally, the comparison between mandibular and maxilla-derived hPDCs identified 438 DEGs; 236 of these genes were up-, and 202 genes were down-regulated in maxilla-derived hPDCs (Figure 5C and Supplementary Table S4).

Next, we performed gene enrichment analysis on the DEGs to capture the biological processes and molecular functions that show significant changes. Genes that show up- and down-regulation in both maxillary and mandibular hPDCs, compared to tibial hPDCs, are involved in skeletal system development, extracellular structure organization, vascular development and related pathways (Supplementary Figures S1, S2). Upregulated genes in tibia showed more significant enrichment in bone development related pathways (Supplementary Figures S1, S2). In the comparison between maxillary and mandibular hPDCs, genes that are up-regulated in mandibular (and also down-regulated in maxillary) were enriched for terms related to skeletal system development, bone remodeling, ossification (Supplementary Figure S3). This reflects the chondrogenic and osteogenic differentiation potential of mandible-derived hPDCs compared to maxillary hPDCs. Down-regulated genes in mandibular (and also up-regulated in maxillary) were involved for terms that are not related to bone development (Supplementary Figure S3).

The list of DEGs also pointed to differences among HOX- and DLX-family genes between craniofacial and tibial hPDCs. The majority of the HOX-family genes showed upregulation in tibial hPDCs. However, some HOX genes (*HOXC8*, *HOXD8*, *HOXC9*, *HOXD9*, and *HOXA10*) showed also higher expression in craniofacial hPDCs (Figure 5D). From the DLX family, *DLX1* was highly expressed in craniofacial and tibial hPDCs, and *DLX5* in mandibular hPDCs.

**FIGURE 6:**
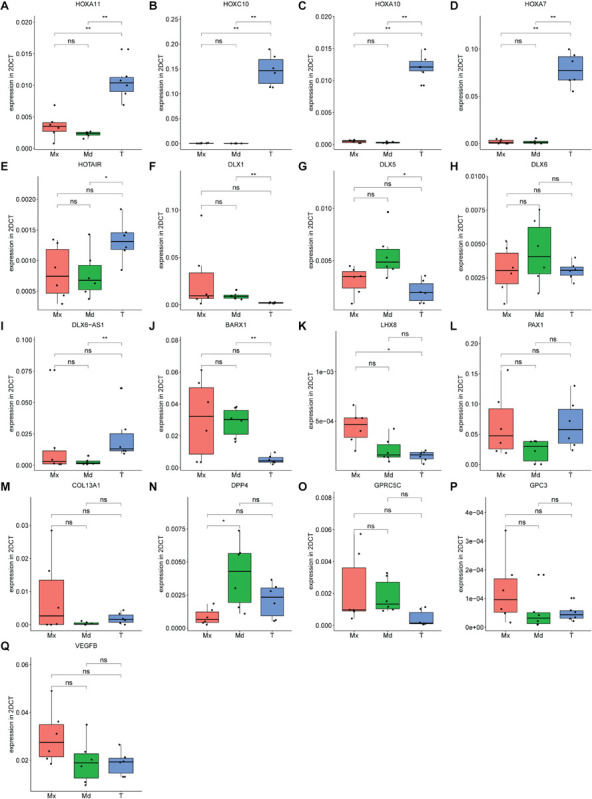
**(A–Q)** Expression level of genes found in top 10 from RNA-sequencing for each comparison, forming in total a top 30 list, confirmed on maxillary (Mx), mandibular (Md) and tibial (T) hPDCs obtained from different donors at PD = 10, *n* = 3 in biological duplicates for each origin corrected for GAPDH. *p ≤ 0.05, **p ≤ 0.01.

The RNA-seq data were validated by RT-qPCR by taking the top-10 DEGs in the 3 comparisons (Figure 5E and Supplementary Table S5). Expression of *HOXA11*, encoding a transcription factor regulating positional cell identity along the anterior-posterior (AP) axis of embryonic segments (Liatsikos et al., 2010; Raines et al., 2015), was elevated in tibial compared to maxillary and mandibular hPDCs (*p* = 0.007 for both; Figure 6A). Also, significantly higher expression values of *HOXA7*, *HOXA10*, *HOXC10*, and the *HOX* transcript antisense RNA (*HOTAIR*) were obtained for tibial hPDCs compared to those from the craniofacial area, which confirmed the RNA-seq results (Figures 6B–E). We conclude that tibia-derived periosteal cells isolated from patients still express genes that are involved in AP patterning in the embryo. *DLX1*, encoding a transcriptional regulator and a downstream target of multiple transforming growth factor beta (TGF-β) family members (Mimura et al., 2016), was more highly expressed in mandibular compared to tibial hPDCs (*p* = 0.007; Figure 6F). *DLX5* is an early-BMP responsive gene and encodes a transcription factor involved in osteoblast differentiation (Li et al., 2008). This gene was more strongly expressed by mandibular compared to tibial hPDCs (*p* = 0.013; Figure 6G). *DLX6*, often proposed as paralog of *DLX1* (Li et al., 2008), did not show any statistically significant difference (Figure 6H), but the antisense RNA encoded by *DLX6* (*DLX6-AS1*), was down-regulated in mandibular compared to tibial hPDCs (*p* = 0.007; Figure 6I). The latter is in accordance with the trend toward a higher expression of *DLX6* in mandibular hPDCs.

*BARX1* is expressed during tooth development and neural crest-derived craniofacial mesenchymal tissues, but is also known for its inhibiting interaction on canonical Wnt signaling (Woo et al., 2011; Lin et al., 2019). Expression of *BARX1* was higher in mandibular compared to tibial hPDCs (*p* = 0.007; Figure 6J). *LHX8* encodes a transcription factor that plays a role in the development of the palate, in oogenesis and neuronal differentiation (Choi et al., 2008; Flandin et al., 2011; Cesario et al., 2015). However, *LHX8* is also known to stimulate the proliferation of MSCs, while a drop in *LHX8* expression level combined with increased levels of BMP2 promotes osteogenesis (Wang et al., 2019). For *LHX8* higher expression was found in maxillary compared to tibial hPDCs (*p* = 0.013; Figure 6K). *PAX1*, a target of Shh signaling that plays a role in axial skeletogenesis (Wallin et al., 1994), showed a trend toward down-regulation in hPDCs originating from the mandible, compared to those from tibia (Figure 6L). *PAX1* also positively regulates the chondrogenic marker genes *SOX5*, *ACAN*, *COL2A1*, and WW domain-containing protein 2 (*WWP2*) (Takimoto et al., 2019). *COL13A1* encodes the alpha-chain of one of the non-fibrillar collagens and has previously been implicated in bone mass and bone formation rate (Ylönen et al., 2005), but did not show any statistically significant differences (Figure 6M). *DPP4* plays an important role in glucose metabolism. Administration of inhibitors against DPP4 results in an increase in bone mass by upregulation of IL-10 expression and subsequent stimulation of chondrocyte hypertrophy (Jung et al., 2013; Wang et al., 2018). *DPP4* was expressed less by maxillary compared to mandibular hPDCs (*p* = 0.015; Figure 6N). *GPC3*, an antagonist of *DPP4* and involved in limb patterning and skeletal development via BMP4 (Paine-Saunders et al., 2000), did not show statistically significant differences, but an opposing trend to *DPP4* (Figure 6P). No statistically significant differences were found for the G-protein coupled receptor *GPRC5C* (Figure 6O). *VEGFB*, involved in angiogenesis and endothelial cell physiology (Hagberg et al., 2010), showed an upregulated trend in maxillary compared to tibial and mandibular hPDCs (Figure 6Q). In total, we could confirm, using RT-qPCR and after Bonferroni-correction, statistically significant differences in expression for 11 out of the 30 DEGs selected from the RNA-seq lists.

### *In vivo* Bone Formation

**FIGURE 7:**
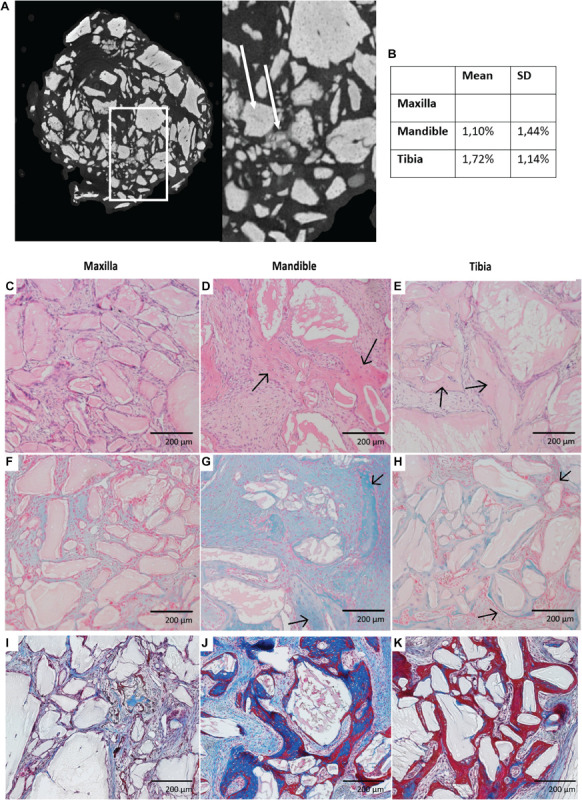
Nano-CT imaging of scaffolds seeded with hPDCs obtained after 8 weeks of ectopic implantation with scaffold grains in light gray (white arrow) and mineralized tissue in dark gray (white dashed arrow). **(A)** Volumetric percentage of mineralized tissue in each cell construct. **(B)** Histological imaging of those cell constructs stained with Hematoxylin & Eosin **(C–E)**, Alcian Blue **(F–H)** with bony tissue pointed by black arrows and Masson’s Trichrome **(I–K)**.

We assessed whether after 8-weeks bone formation in scaffolds seeded with tibial hPDCs did occur; for this, we used Nano-CT analysis of ectopically implanted scaffolds (Figures 7A,B). No *in vivo* mineralized tissue was detected with maxilla-derived hPDCs, whereas it was present, as a positive control, in constructs seeded with tibial hPDCs (*p* = 0.04326) at an average of 0.0736 mm^3^ bone tissue (Figures 7A,B). Importantly, bone formation by mandibular hPDCs was detected in 6 out of the 12 retrieved constructs, averaging 0.06 mm^3^ bone tissue in those 12 constructs. Staining with hematoxylin/eosin (HE) confirmed that the mineralized tissue seen on CT-scans was indeed bone tissue (Figures 7C–E). Staining of cartilage with Alcian Blue showed more staining in scaffolds seeded with mandibular hPDCs (Figures F–H). Staining for bone tissue with Masson’s Trichrome confirmed the results obtained from both HE and Alcian Blue staining (Figures I–K).

## Discussion

Research toward cell-based bone tissue-engineering recently focused strongly on the properties of SSPCs and proposed hPDCs as candidate cells (Roberts et al., 2015; Bolander et al., 2020). We have documented the differences between PDCs obtained from different anatomical locations (maxilla, mandible and tibia, respectively) *in vitro* and also *in vivo*. FACS on *in vitro* expanded cells scored them as CD73+, CD164+, and PDPN+, each marker associated with SSPC identity, whereas they were negative for hematopoietic lineage markers. The latter is important, for the presence of one or more hematopoietic markers on SSPCs was shown by others to underlie further cellular differentiation accompanied by loss of stem cell state (van Lochem et al., 2004). We concluded that 88% of our *in vitro* expanded craniofacial cells fulfilled the applied SSPC criteria, and 62% of our *in vitro* expanded tibial hPDCs, but also acknowledged the limited relevance of these markers for stem cell function (Ambrosi et al., 2019). Our hPDCs were able to proliferate *in vitro* at comparable rates to each other, they did not display senescence during 9 weeks of culture, and were able to differentiate at CPD∼8 to osteogenic, chondrogenic and adipogenic lineages. *ALPL* and *BGLAP* displayed higher mRNA expression *in vitro* cultured hPDCs obtained from maxilla compared to tibia, which could underlie the higher *in vitro* osteogenic potential of maxilla-derived hPDCs. For *in vivo* endochondral bone formation, the mandibular hPDCs showed a higher potential, possibly due to higher *SOX9* expression by these cells *in vitro*, again compared to tibial hPDCs. The steady-state mRNA levels of *VEGFR-1*, encoding a receptor that is normally upregulated during vascularization in healing of fractured bones, were highest in maxillary hPDCs compared to the other ones. This can be of interest when scaling-up tissue engineering constructs that have diffusion limits of oxygen and nutrients, since cell death or apoptosis in the center of the implant has been reported if good vascularization is not achieved quickly (Potier et al., 2007; Giannoni et al., 2010).

RNA-seq and further validation by RT-qPCR on separate *in vitro* cultured samples thus reveal notable differences in gene expression between hPDCs obtained from the three sites. These are represented mostly by the higher expression of HOX and DLX family genes steering skeletal system and other connective tissue development, but also co-determine extracellular matrix organization, collagen formation, and responses to growth factors. Overall, these sets of genes are irrefutably relevant to *in vivo* bone regeneration and form a solid basis for future experiments that aim at improving bone tissue engineering applications. *HOXA7*, *HOXA10*, *HOXC10*, and *HOTAIR* were each upregulated in tibial hPDCs compared to those from the craniofacial area. Hence, tibia-derived periosteal cells isolated from adult patients still express genes that are involved in several other processes, including anterior-posterior patterning in the embryo, or these genes also have additional functions in cell differentiation. Leucht et al. (2008) grafted periosteum from the tibia onto craniofacial bone defects (and vice versa) and demonstrated that *HOXA11*^+^ cells maintain their HOX status after changing the environment, but *HOXA11*-negative cells can start to express *HOXA11* after changing the environment. In this study, the change of environment, which *in vitro* expansion is, could explain the presence of low expression of HOX genes by hPDCs obtained from the maxilla and mandible. *DLX1* was expressed at a higher level in maxilla-derived hPDCs. This could explain the higher osteogenic potential of these cells *in vitro*. *DLX5* acts as an early BMP-responsive transcriptional activator driving osteoblastic differentiation of cells, while *DLX6* has been proposed as paralog of *DLX1* (Li et al., 2008). These results suggest favoring the use of mandibular hPDCs in craniofacial applications, since *DLX5* and *DLX6* where both up-regulated in these cells. However, *PAX1*, a target of Shh signaling, but also positively regulating the chondrogenic effector or marker genes *SOX5*, *ACAN*, *COL2A1*, and *WWP2* (Wallin et al., 1994; Takimoto et al., 2019), was expressed at higher levels in tibial hPDCs. Taken together, hPDCs obtained from maxilla, mandible and tibia showed different gene/mRNA expression profiles, including of relevant effector or marker genes, which could lead to different direct or endochondral bone forming properties. This could be exploited in bone tissue engineering strategies for various anatomical locations. For a number of selected genes, based on RNA-seq, we also could not reproduce the results by RT-qPCR. We hypothesize that this is due to our use of different donors throughout these comparisons, which made the validation more stringent.

Analysis of hPDC-seeded scaffolds showed bone formation 8 weeks after ectopic implantation when seeded with tibial and mandibular cells, but unexpectedly not with maxilla-derived hPDCs. For the latter, cells show *in vitro* upregulation of osteogenic genes compared to tibia-derived cells. We hypothesize that maxilla-derived periosteal cells are less prone for endochondral bone formation when the cells are not stimulated by growth factors. Also local environmental cues influence the ability of these cells to form bone, as demonstrated previously through periosteal grafting experiments (Leucht et al., 2008). In addition, the choice of the carrier or any coincidence in view of the generally observed biological variability of the *in vivo* ectopic bone formation assays, could account for the documented difference (Ji et al., 2018).

To our knowledge, *in vivo* results of ectopic bone formation using *in vitro* expanded maxilla-derived periosteal cells have not been reported yet. Additional work is needed to investigate if priming of hPDCs prior to implantation could be optimized for craniofacial-derived hPDCs, as was shown for tibial hPDCs (Bolander et al., 2016). The effects of priming tibia-derived periosteal cells with different concentrations of calcium in the medium on their bone-forming capacities *in vivo*, and when using calcium phosphate scaffolds, have been documented previously (Chai et al., 2012). Mandibular periosteal cells, obtained from the region near the wisdom tooth, yield more bone tissue *in vivo* when co-cultured with CD34^+^ endothelial progenitors obtained from the umbilical cord, while a trend toward a better osteogenic potential of mandibular periosteum-derived cells cultured using cyclic tension force has also been reported (Lee et al., 2014, 2017). For tibia-derived periosteal cells, the effects of different calcium phosphate scaffolds on *in vivo* bone formation capacities of such cells was also reported (Kerckhofs et al., 2016). Others have stated the importance of the combination of mesenchymal lineage-derived cells with type of scaffold (Mattioli-Belmonte et al., 2015), but this has not yet been explored with maxilla and/or mandible-derived cells. Furthermore, many features of scaffolds contribute to the *in vivo* outcome, including bio-compatibility, porosity, pore size, surface properties, osteo-inductivity and mechanical properties (Salgado et al., 2004). In addition, the predictability and robustness of the *in vivo* formed bone construct as well as possible differences in the speed of bone formation, and bone formation in an orthotopic environment altogether, should also be evaluated and/or improved in order to prepare translation toward the clinic (Geris and Papantoniou, 2019).

We hypothesized that location and tissue of origin are relevant for successful bone repair when using *in vitro* expanded cell-based tissue engineering constructs. hPDCs from different anatomical locations may be interesting sources, but would display different properties and possibly differences in outcome with regard to bone healing *in vivo*. Therefore, we focused on documenting (dis)similarities between *in vitro* expanded periosteum-derived cells from tibia, maxilla and mandible. Progenitor cells from different anatomical sources indeed differ in their transcriptomic signature and *in vivo* differentiation potential (Sacchetti et al., 2016). We have shown that *in vitro* expanded hPDCs obtained from these respective sites do not differ in their capacities to proliferate *in vitro* under the experimental conditions used. However, the cells showed differences in gene expression profiles and *in vivo* bone formation. So, different properties, including embryonic origin and (epi)genetic priming laid down within functionally similar cell types, located at different anatomical sites, can be retained during *ex vivo* expansion. This then influences the functional outcome, here demonstrated by different skeletogenic potential of SSPCs. It remains to be investigated if these observed differences are critical for clinical outcomes, including remodeling of tissue over time, and for which indications.

## Data Availability Statement

The datasets presented in this study can be found in online repositories. The names of the repository/repositories and accession number(s) can be found below: https://www.ncbi.nlm.nih.gov/geo/, GSE149167.

## Ethics Statement

The studies involving human participants were reviewed and approved by Ethics Committee Research UZ/KU Leuven. Written informed consent to participate in this study was provided by the participants or their legal guardian. The animal study was reviewed and approved by KU Leuven Animal Ethics Committee.

## Author Contributions

LG contributed to conception, design, and data acquisition, drafted and critically revised the manuscript. TH contributed to the data analysis and critically revised the manuscript. MM and FL contributed to conception, design supported in case of problems, and critically revised the manuscript. CP contributed to conception and critically revised the manuscript. WI contributed to data acquisition, initial data analysis, and critically revised the manuscript. DH and EM contributed to conception, design, data acquisition, data analysis, interpretation of results, and critically revised the manuscript. LG contributed to conception, supported in case of problems and critically revised the manuscript. All authors gave final approval and agree to be accountable for all aspects of the work.

## Conflict of Interest

The authors declare that the research was conducted in the absence of any commercial or financial relationships that could be construed as a potential conflict of interest.

## Supplementary Material

Supplementary Figure 1 Gene ontology terms for up- and downregulated genes between hPDCs obtained from maxilla and tibia.

Supplementary Figure 2 Gene ontology terms for up- and downregulated genes between hPDCs obtained from mandible and tibia.

Supplementary Figure 3 Gene ontology terms for up- and downregulated genes between hPDCs obtained from maxilla and mandible.

Supplementary Table 1 Primer sequences used for RT-qPCR.

Supplementary Table 2 List of up- and downregulated genes of hPDCs obtained from maxilla compared to tibial hPDCs.

Supplementary Table 3 List of up- and downregulated genes of hPDCs obtained from mandible compared to tibial hPDCs.

Supplementary Table 4 List of up- and downregulated genes of hPDCs obtained from mandible compared to maxillary hPDCs.

Supplementary Table 5 Primer sequences of selected top-30 genes after RNA-sequencing, used for RT-qPCR.
