# Supplementary figures and images for: The Bone-Forming Properties of Periosteum-Derived Cells Differ Between Harvest Sites
Source: Front Cell Dev Biol. 2020 Nov 25;8:554984. doi: 10.3389/fcell.2020.554984 (PMC7723972; doi:10.3389/fcell.2020.554984)

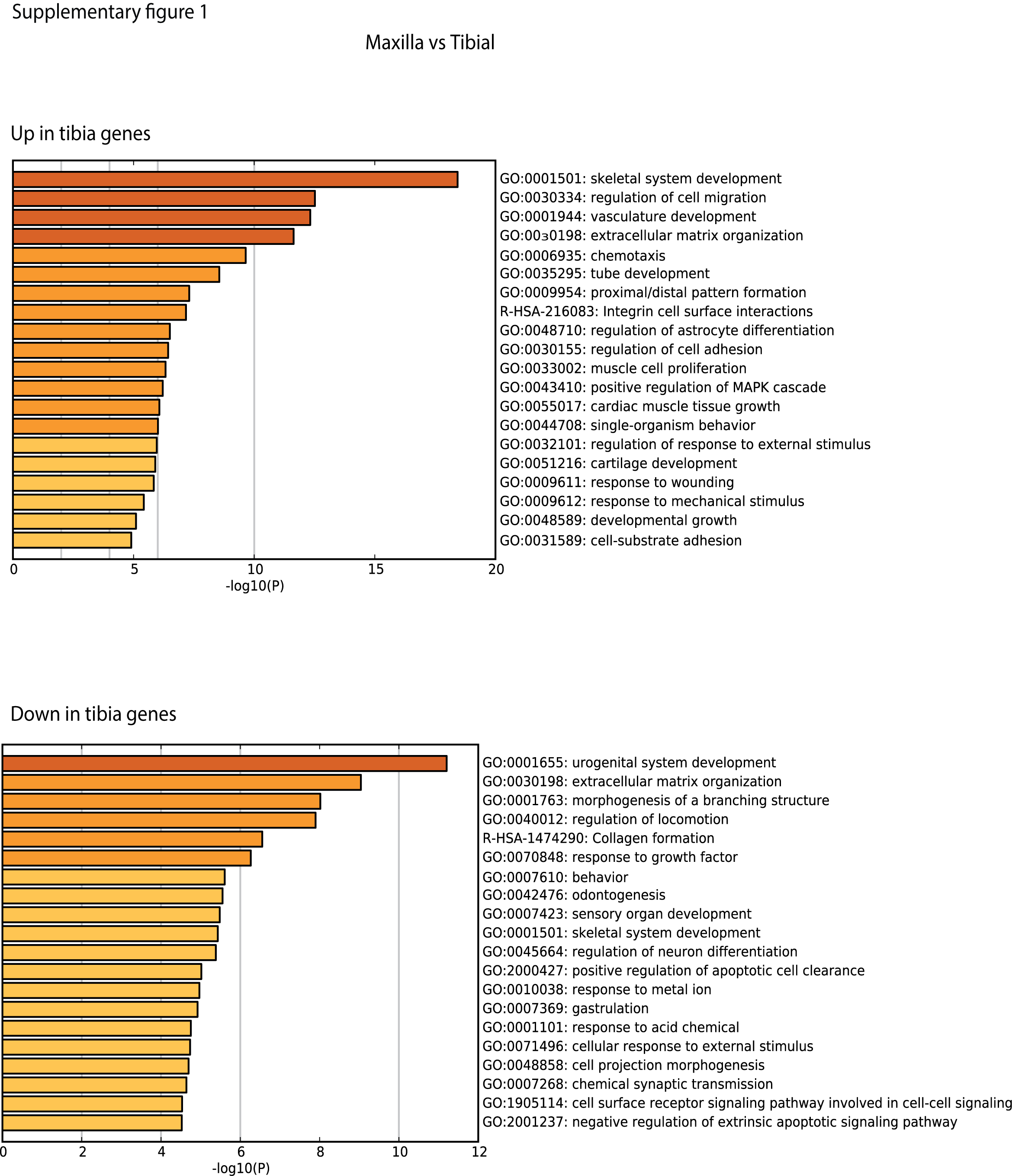

Supplement: Supplementary Figure 1 — Gene ontology terms for up- and downregulated genes between hPDCs obtained from maxilla and tibia. [file Image_1.TIF]

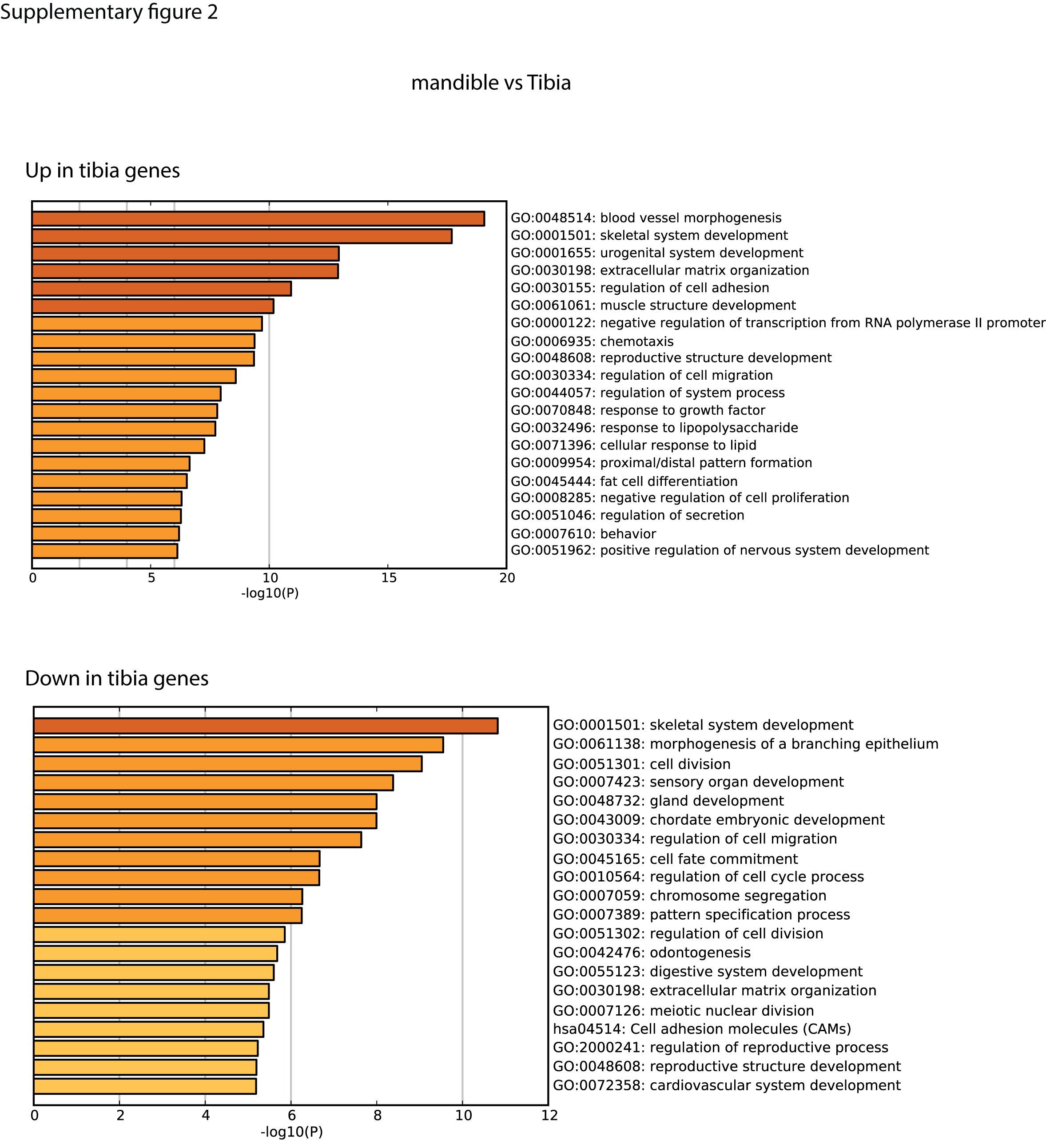

Supplement: Supplementary Figure 2 — Gene ontology terms for up- and downregulated genes between hPDCs obtained from mandible and tibia. [file Image_2.TIF]

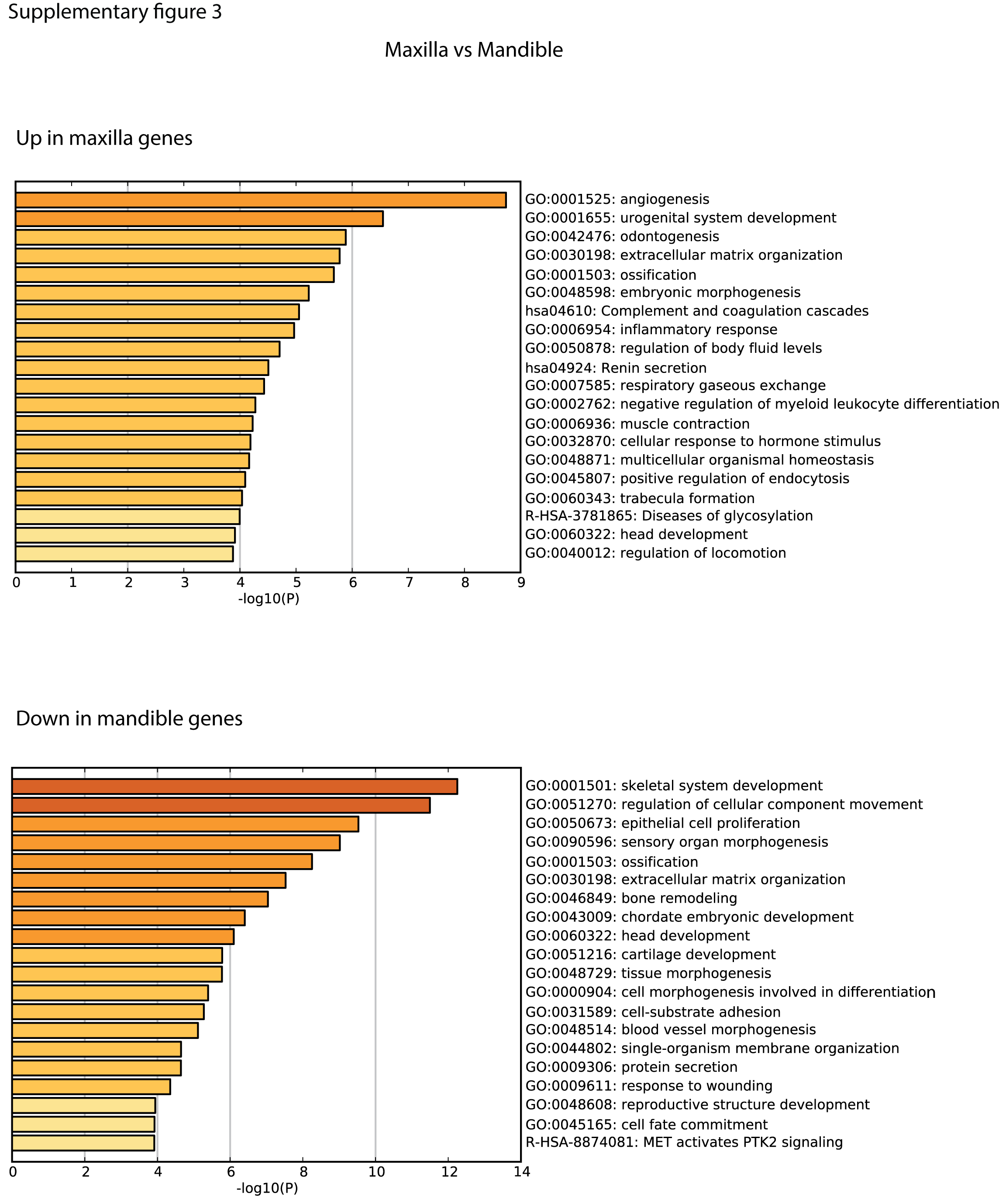

Supplement: Supplementary Figure 3 — Gene ontology terms for up- and downregulated genes between hPDCs obtained from maxilla and mandible. [file Image_3.TIF]
