# Supplementary material for: The Bone-Forming Properties of Periosteum-Derived Cells Differ Between Harvest Sites
Source: Front Cell Dev Biol. 2020 Nov 25;8:554984. doi: 10.3389/fcell.2020.554984 (PMC7723972; doi:10.3389/fcell.2020.554984)
Supplement: Supplementary Table 1 — Primer sequences used for RT-qPCR. [file Table_1.DOCX]

**Table S1: Primer sequences used for RT-qPCR.**

| Gene | Forward | Reverse |
| --- | --- | --- |
| *ALPL* | GCTTCAAACCGAGATACAAGCA | GCTCGAAGAGACCCAATAGGTAGT |
| *BGLAP* | GTGCAGCCTTTGTGTCCAA | GCTCACACACCTCCCTCCT |
| *COL10A1* | ACGATACCAAATGCCCACAG | GTGGACCAGGAGTACCTTGC |
| *GAPDH* | CGATGCTGGCGCTGAGTAC | CGTTCAGCTCAGGGATGACC |
| *PPARG* | GCCAAGCTGCTCCAGAAAAT | TGATCACCTGCAGTAGCTGCA |
| *SOX9* | TGGAGACTTCTGAACGAGAGC | CGTTCTTCACCCACTTCCTC |
| *SPP1* | ACTGATTTTCCCACGGACCT | TCAGGGTACTGGATGTCAGG |
| *VEGFR* | ATTTGTGATTTTGGCCTTGC | CAGGCTCATGAACTTGAAAGC |
| *ACAN* | GTCTCACTGCCCAACTAC | GGAACACGATGCCTTTCAC |
| *RUNX2* | CGCATTCCTCATCCCAGTAT | GCCTGGGGTCTGTAATCTGA |
