# Supplementary material for: The Bone-Forming Properties of Periosteum-Derived Cells Differ Between Harvest Sites
Source: Front Cell Dev Biol. 2020 Nov 25;8:554984. doi: 10.3389/fcell.2020.554984 (PMC7723972; doi:10.3389/fcell.2020.554984)
Supplement: Supplementary Table 5 — Primer sequences of selected top-30 genes after RNA-sequencing, used for RT-qPCR. [file Table_5.DOCX]

**Table S5: Primer sequences of selected top-30 genes after RNA-sequencing, used for RT-qPCR.**

| Gene | Forward code | Reverse code |
| --- | --- | --- |
| *HOXC10* | CGGATAACGAAGCGAAAGAGGAG | GCGCTCTCGCGTCAAATACA |
| *HOXA10* | AGGATTCCCTGGGCAATTCCAAA | TTGTCTGTCCGTGAGGTGGA |
| *HOXA11* | TTTGATGAGCGTGGTCCCTG | AGTATGTCATTGGGCGCGAA |
| *HOXC9* | CCCCAGTAAGTTGGGAGCAAT | CCGACGGTCCCTGGTTAAAT |
| *HOXA7* | GGAGTTCCACTTCAACCGCT | CGGACCTTCGTCCTTATGCTC |
| *HOTAIR* | GCCAGTACCGACCTGGTAGA | GTCTGTGAGTGCCCGTCTTG |
| *TMEM255B* | CGTCCTCATAGTCACCGTCG | GCCACCAGCATTTGCCTTC |
| *DPP4* | GCTCGGCGCTCACTAATGTT | CACGGTGTCTTCATCGTCGG |
| *DLX5* | CACGGCTACTGCTCTCCTAC | CTTCTTTCTCTGGCTGGTTGGTG |
| *DLX6* | GAGGGGACGACACAGATCA | GTTCGGCTCTCTCTGGAAGG |
| *DLX6-AS1* | GGAGGATTCTGTGTGGGGTTG | ATGGGAGCACTCAGCCTACC |
| *BDKRB1* | GCCCCCTCTAGAGCTCCAAT | AAAGGTTCCCTAGGAGGCCG |
| *LRRC15* | GAGCTGCTGATGTGTCCCTAGC | GCCCACCAGCAAAAGGAGATAA |
| *AFF2* | TCTTGCGGGAGATGACCCATT | GACACTGACTTTGTAGAAGCTCTGG |
| *COL13A1* | CCACACCGGGAGTGCCTAAG | CCTGGAACGTCCGCCTTTTT |
| *STAT4* | TGGGTGGGAACTGACCCAAG | AGACATGCTAGCGCTCTCTCAG |
| *DLX1* | CGCTTCAATGGCAAGGGAAA | ATCTTGACCTGAGTCTGTGTG |
| *TFAP2C* | CTCCACGACATGCCTCACCA | TGGAAATGGGACCTTTGCGAAT |
| *SMAGP* | TTCAAAAGGACGCGCGGAG | GGGGTGGTCATCAGTTCTTCTCT |
| *HOMER2* | TGGGAGAACAGCCCATCT | TATGATCACCTTGGCTCCGTC |
| *VEGFB* | TATACTCGCGCTACCTGCCA | TGAGGATCTGCATCCGGACTT |
| *BARX1* | CAAAGCCAAGAAGGGGCGTC | ATCTATTCTGTCCGGCGTGGAA |
| *LHX8* | CTGGACCACTTGTGCTGGAGT | GGAAGCGTTTCCAGTCAAGCC |
| *PAX1* | TGCGCAAGGTCCTCCTCTG | GGAGGCTTCCTTCTCGGCT |
| *HCN1* | CTTTGGAGAGATTTGCCTGCT | AGTCGGTCAATGGCAACTGT |
| *SLC1A7* | GGCTCTCATGCGTGGAATGG | TAATTTCCTGTGGTGAGAGGCG |
| *PALMD* | AGACTCCAGGCCATCACAGATAA | TTCCTGTTCTTTTCCGCTGCT |
| *GPC3* | ACCACCACTGAAACTGAGAAGAA | TTGGCGTTGTTGAGAATGGGC |
| *GPRC5C* | TGCACAAAGTTCCGTCCGAAG | GTCCCACACGTAGGGGTTTCT |
| *TMEM150C* | AGATCCCACCCCTTCCTGA | CCATGCCTGTACCACTGCT |
